# Persistent T Cell Repertoire Perturbation and T Cell Activation in HIV After Long Term Treatment

**DOI:** 10.3389/fimmu.2021.634489

**Published:** 2021-02-25

**Authors:** Carolin T. Turner, James Brown, Emily Shaw, Imran Uddin, Evdokia Tsaliki, Jennifer K. Roe, Gabriele Pollara, Yuxin Sun, James M. Heather, Marc Lipman, Benny Chain, Mahdad Noursadeghi

**Affiliations:** ^1^ Division of Infection and Immunity, University College London, London, United Kingdom; ^2^ Departments of HIV and Respiratory Medicine, Royal Free London NHS Foundation Trust, London, United Kingdom; ^3^ UCL Respiratory, Division of Medicine, University College London, London, United Kingdom

**Keywords:** human immunodeficiency virus, T cell repertoire, blood transcriptome, antiretroviral therapy, people living with HIV, chronic inflammation, T cell activation

## Abstract

**Objective:**

In people living with HIV (PLHIV), we sought to test the hypothesis that long term anti-retroviral therapy restores the normal T cell repertoire, and investigate the functional relationship of residual repertoire abnormalities to persistent immune system dysregulation.

**Methods:**

We conducted a case-control study in PLHIV and HIV-negative volunteers, of circulating T cell receptor repertoires and whole blood transcriptomes by RNA sequencing, complemented by metadata from routinely collected health care records.

**Results:**

T cell receptor sequencing revealed persistent abnormalities in the clonal T cell repertoire of PLHIV, characterized by reduced repertoire diversity and oligoclonal T cell expansion correlated with elevated CD8 T cell counts. We found no evidence that these expansions were driven by cytomegalovirus or another common antigen. Increased frequency of long CDR3 sequences and reduced frequency of public sequences among the expanded clones implicated abnormal thymic selection as a contributing factor. These abnormalities in the repertoire correlated with systems level evidence of persistent T cell activation in genome-wide blood transcriptomes.

**Conclusions:**

The diversity of T cell receptor repertoires in PLHIV on long term anti-retroviral therapy remains significantly depleted, and skewed by idiosyncratic clones, partly attributable to altered thymic output and associated with T cell mediated chronic immune activation. Further investigation of thymic function and the antigenic drivers of T cell clonal selection in PLHIV are critical to efforts to fully re-establish normal immune function.

## Introduction

Effective anti-retroviral therapy (ART) suppresses circulating human immunodeficiency virus (HIV) to undetectable levels, and has transformed the health of people living with HIV (PLHIV) by abating progression to AIDS and allowing near-normal life expectancy (1–3). However, PLHIV still experience greater morbidity due to chronic cardiovascular and respiratory disease, cancer, and infection (4–6). Some of this is attributed to exposures such as tobacco smoking (7, 8) and ART-related toxicity (9), but predictors of clinical outcome in this context also include circulating markers of inflammation such as pro-inflammatory cytokines (IL6), acute phase proteins (CRP, fibrinogen) and markers of leukocyte activation (soluble CD14 and CD163), suggesting persistent immune dysregulation in PLHIV despite effective ART (10, 11). Enrichment of pro-inflammatory states has also been reflected in transcriptional studies of blood monocytes (12), myeloid dendritic cells (13) and CD4 T cells (14) from ART-treated PLHIV. The immunological pathways that underpin these observations are not fully understood. They are partly attributed to low-level HIV replication in specific anatomical niches (15–18), and to ongoing translocation of microbial products (19, 20) as a result of reduced gastrointestinal barrier integrity, particularly related to a reduction in mucosal-associated invariant T cells (21, 22) and Th17 cells (23, 24). Importantly, HIV-associated changes in circulating T cell numbers may also persist despite ART. This includes incomplete reversal of CD4 T cell depletion (25) and relatively elevated numbers of CD8 T cell counts (26, 27) leading to a low CD4:CD8 ratio as a persistent immunological hallmark of PLHIV (28–31).

We and others have previously established that untreated HIV infection leads to a profound reduction in the diversity of T cell clones and oligoclonal T cell expansion reflected by T cell receptor (TCR) sequence analysis, resulting in skewed and highly idiosyncratic repertoires (32–37). Effective ART over three months that reduced HIV viral load and increased circulating CD4 T cells, did not reverse the overall changes in the T cell repertoire (37). The effect of long-term ART on the T cell clonal repertoire is not known. In the present study, we sought to extend our understanding of chronic immunological dysfunction in PLHIV on long-term ART. We used high-throughput TCR sequencing to identify persistent abnormalities in the clonal T cell repertoire, and genome-wide whole blood transcriptional profiling to evaluate whether changes in the repertoire are associated with changes in expression of gene sets indicative of altered immune function.

## Methods

### Ethical Approvals

This study was approved by the London Hampstead Research Ethics Committee (14/LO/1409) and registered with the ISRCTN registry (http://www.isrctn.com/ISRCTN38386321). All participants provided written informed consent.

### Study Population

Samples were collected as part of a prospective cohort study of PLHIV attending routine ambulatory HIV care at the Royal Free London NHS Foundation Trust, and of HIV-negative healthy controls (HC) recruited from Sexual Health and General Practice clinics. At recruitment, blood samples were collected in Tempus tubes for cryo-storage prior to RNA extraction. Clinical and laboratory data including lymphocyte subset counts and HIV viral load measurements were obtained from participants’ hospital records. In addition, each participant completed the health-related quality of life EuroQol 5D (EQ5D) questionnaire, where a lower score represents a worse outcome (38), and the St George’s Respiratory Questionnaire (SGRQ), a participant reported measure of respiratory health status, where a higher score represents a worse outcome (39). To minimise confounding by differences in ethnicity or gender, only samples from white European male participants were utilised for the present study.

### T Cell Receptor Repertoire Sequencing and Data Processing

Total RNA was extracted from blood and subjected to next generation sequencing of alpha and beta chains of the TCR repertoire on the Illumina NextSeq platform, using an established quantitative TCR sequencing pipeline that integrates experimental library preparation and computational analysis with Decombinator (version 3.1) (40, 41). All software is freely available at https://github.com/JamieHeather/Decombinator. For each TCR, Decombinator specifies the V and J gene used, the number of V and J gene deletions (relative to the germline sequence), and the nucleotide insert sequence between the end of the deleted V and J genes. Unless otherwise stated, analyses were performed with TCRs defined by the amino acid sequence of the encoded complementary determining region 3 (CDR3), running from the last conserved cysteine in the V to the conserved phenylalanine in the FGXG motif in the J gene.

### Quantifying the T Cell Receptor Repertoire

The T cell receptor repertoire of an individual is uniquely defined by the number of different TCR sequences which it contains, and their relative abundance. Many different algorithms have been developed which seek to capture this information in a single metric, so that different repertoires can be compared. In this study we use three metrics commonly used in studies of the repertoire. Repertoire richness is simply the total number of distinct TCRs present in the repertoire, irrespective of their abundance. The Gini coefficient captures repertoire inequality, ranging from zero (all TCRs are present at equal abundance) to one (repertoire contains only one TCR). The Shannon entropy, like richness, is a measure of repertoire diversity. In contrast to richness, it gives weight to sequences on the basis of their abundance. TCR-rich samples with a more even repertoire distribution yield a higher Shannon index value. Gini and Shannon indices were calculated as previously described (37, 42). All three metrics are influenced by sample size, since in practice we estimate the true TCR repertoire on the basis of a small sample of the whole repertoire. To account for different sequencing depth between samples, therefore, these indices were also calculated by randomly sub-sampling individual repertoires 100 times to the same number of total sequences, and calculating the average metric for each individual. The customized python script is available at https://github.com/innate2adaptive/Decombinator/blob/master/SupplementaryScripts/RandomlySample.py.

### CMV Status Prediction and Quantitation of HIV, CMV and EBV-Reactive T Cell Receptor Sequences

CMV status of participants was predicted based on the presence or absence of CMV-targeting CDR3 beta sequences that are used in the context of the correct V and J genes, following a previously described statistical classification approach (43). Alpha and beta CDR3s among the 100 most expanded sequences were annotated as HIV, CMV or EBV-reactive if they were listed as sequences known to target these viruses in the context of the correct chain on the VDJdb database (https://vdjdb.cdr3.net/; accessed 26/11/2019) (44).

### Intra-Individual Similarity of T Cell Receptor Sequences

To measure similarity between pairs of CDR3s within individual repertoires, the Levenshtein distance (the minimum number of single-character edits required to turn one string into another) was calculated using the R package stringdist. The pairwise similarity matrix was then converted into a network diagram (R package igraph), where two CDR3 sequences (nodes) were connected (by an edge) if they differed by a Levenshtein distance of one. A cluster was defined as a set of four or more nodes that are connected to each other by any number of edges. Frequency distributions of Levenshtein distances (and CDR3 lengths) were visualized with heatmaps created in Morpheus (https://software.broadinstitute.org/morpheus).

### Inter-Individual Sharing of Identical T Cell Receptor Sequences

Pairwise assessments of the overlap of identical CDR3 sequences between individuals were made with the Jaccard index, which is the number of TCRs shared between two repertories counting each unique TCR only once (i.e. independent of abundance). Sharing of identical CDR3 sequences among PLHIV or HC was further determined by counting in how many individuals a given CDR3 appeared. To correct for the larger size of the group of PLHIV (n=26 versus n=12 HC), this analysis was performed in 100 random samples of 12 out of the 26 PLHIV samples available.

### Blood RNA Sequencing and Data Processing

Genome wide mRNA sequencing of the same samples subjected to TCR repertoire analysis was performed as previously described (45), resulting in a median of 26 million (range 21–31 million) 41 bp paired-end reads per sample. RNAseq data were mapped to the reference transcriptome (Ensembl Human GRCh38 release 95) using Kallisto (46). The transcript-level output counts and transcripts per million (TPM) values were summed on gene level and annotated with Ensembl gene ID, gene name, and gene biotype using the R/Bioconductor packages tximport and BioMart (47, 48). Downstream analyses were restricted to gene biotypes with selected BioMart annotations (protein coding, IG_C_gene, IG_D_gene, IG_J_gene, IG_V_gene, TR_C_gene, TR_D_gene, TR_J_gene, TR_V_gene), resulting in 23,289 Ensembl gene IDs. Differential gene expression was analyzed with DeSeq2 and SARTools packages (49), using a false discovery rate (FDR) <0.05. For all other analyses, gene expression was represented by log_2_-transformed TPM values, following the addition of a pseudocount of 0.001.

### Molecular Degree of Perturbation

The modified MDP was derived using the mdp R/Bioconductor package (50). This provides a single measure of the quantitative difference between a given transcriptome and a standard reference representative of a healthy state. For each participants’ transcriptome the MDP was represented by the median Z score >2 of individual gene expression values calculated by subtracting the mean and dividing by the standard deviation of gene expression values among the 12 healthy controls used as the standard reference.

### Ingenuity and Reactome Pathway Analysis

Ingenuity pathway analysis (Qiagen) was used to identify the interactome of differentially expressed genes, and to probe interacting genes further for predicted upstream regulators. The ten most significant upstream regulators with activation Z-scores >2 were visualized as a network in Gephi v0.9.2. Reactome pathway enrichment of differentially expressed, interacting genes was analyzed with the XGR R package (51). For visualization, 20 pathway groups were identified by hierarchical clustering of Jaccard indices to quantify similarity between the gene compositions of each pathway. For each group, the pathway with the largest total number of genes was then selected to provide a representative annotation.

### Transcriptional Modules

The HIV module was derived from blood microarray data of an independent set of healthy controls and HIV patients before and after three months of anti-retroviral therapy. Transcriptional profiling by Agilent microarrays (SurePrint G3 Human Gene Expression v3 8×60K or Human Gene Expression v2 4×44K platform) and subsequent data processing were undertaken as previously described (52). Probes that were represented on both microarray platforms were retained for analysis. Probe annotations were downloaded from Agilent’s eArray web portal, and duplicate gene names removed to retain the gene name with highest average expression across all samples. Differential expression among the resulting 14,706 gene names was analyzed using Mann-Whitney tests in MultiExperiment Viewer v4.9 (http://www.tm4.org/mev.html) with FDR <0.05. Genes with a median expression value ≥2−fold in the untreated HIV patient group compared to healthy controls were included in the HIV module (**Supplementary File 1**). The derivation and validation of the macrophage type 1 IFN module has previously been published (53). The T helper cell type 1 IFN module was derived from published transcriptomes of CD4 T cells cultured in the presence of IFNα and stimulated with anti-CD3 and anti-CD28 (dataset GSE54627) (54). Genes over-expressed more than 1.5-fold compared to Th1, Th2, or Th17 polarized CD4 T cells by paired t-test with p <0.05 were included in the T cell type 1 IFN module (**Supplementary File 2**). To quantify CD4 T cell frequency we used the ‘Cluster0127: High in CD4 T cells’ module by Mabbott et al. (55), and to quantify CD8 T cell frequency we used the ‘CD8’ module by Watkins et al. (56). In a comparison with 15 other CD4 T cell modules and 7 other CD8 T cell modules, both these modules achieved the highest sensitivity and specificity for their target cell type, when applied to transcriptomic data from other immune cells across multiple datasets, as quantified by the modular discrimination index (MDI) score (57). Gene module scores were calculated as mean expression of the constituent gene names in each module. Where duplicate gene names were present in the RNAseq data, the highest log_2_ TPM value was used for each sample.

### Statistics

Analyses were performed in R (version 3.6.0) or python (version 2.7.15) as described above. Statistical differences were assessed in GraphPad Prism (version 8.3.1) using the tests stated in the text and Figure legends, and considered significant for p <0.05.

## Results

### T Cell Receptor Repertoires Remain Disturbed in People Living With HIV Despite Effective Anti-Retroviral Therapy

We used a cross-sectional study design to test the hypothesis that the T cell clonal repertoire returns to normal after long-term ART by comparison of 26 PLHIV on long-term ART and 12 HIV-negative controls among white European men, with comparable age range and health-related questionnaire scores (**Table 1**). PLHIV were on ART for a median of 8.5 years (interquartile range (IQR) 3–16 years). They had undetectable plasma HIV RNA (<40 copies/ml) and median circulating CD4 counts of 703 cells/µl (IQR, 491–841 cells/µl).

**Table 1:** Cohort description.

		Healthy controls (HC)	People living with HIV (PLHIV)
Subjects		12	26
Age, years		50 (48–55)	51.5 (47–56)
Male sex		12 (100%)	26 (100%)
White British/Irish		12 (100%)	26 (100%)
Tobacco smoking	Current	3 (25%)	5 (19%)
	Ex-smoker	7 (58%)	10 (38%)
	Never	2 (17%)	11 (42%)
EQ5D index		0.96 (0.85–1.00)	0.89 (0.78–1.00)
SGRQ score		6.2 (1.9–10.8)	11.6 (3.7–35.1)
ART duration, years		–	8.5 (3–16)
CD4 pre-ART		–	279 (155–414)
CD4 at point of sampling		–	703 (491–841)
CD8 at point of sampling		–	984 (694–1362)
CD4/CD8 at point of sampling		–	0.77 (0.50–0.86)
CMV serology			
Positive Negative No data		– – 12	11 2 13
Predicted CMV status ^%^			
Positive Negative		10 2	23 3

Data are shown as median (interquartile range) or number (percentage). ART, anti-retroviral therapy; EQ5D, EuroQuol 5-dimensions (generic health-related questionnaire); SGRQ, St. George’s Respiratory Questionnaire; CMV, cytomegalovirus. ^%^CMV status was predicted based on T cell receptor beta chain sequences that are associated with CMV seropositivity (43). Missing data: CD4 pre-ART (n = 1 PLHIV), SGRQ (n = 2 HC, n = 4 PLHIV).

**Figure 1:**
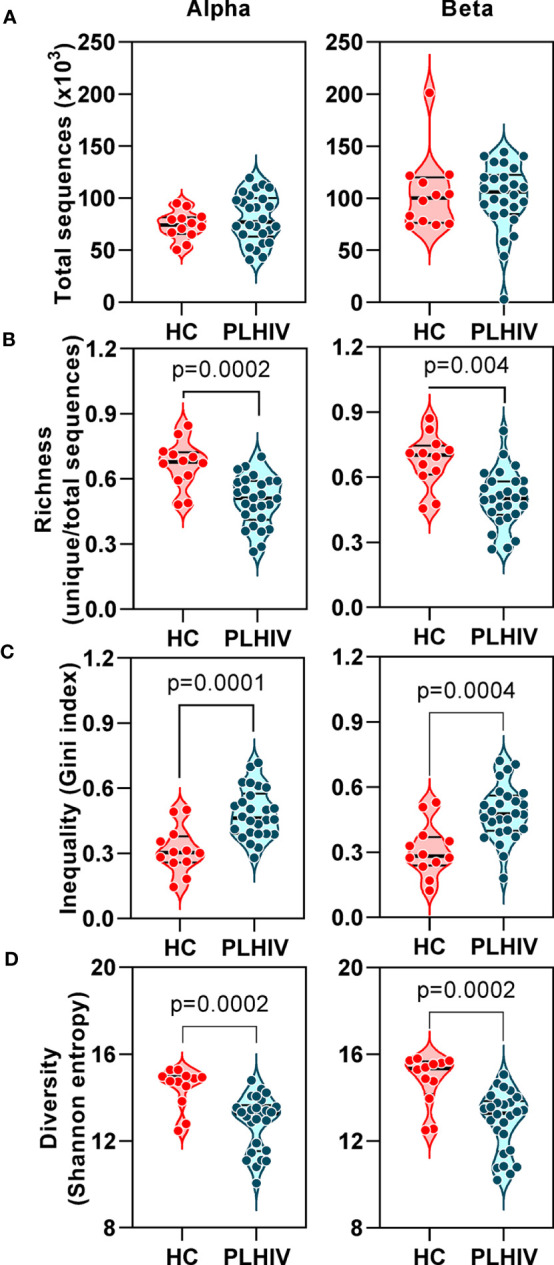
T cell receptor alpha and beta chain sequence repertoires remain disturbed in people living with HIV (PLHIV) despite long term effective anti-retroviral therapy. **(A)** Total number of CDR3 sequences. **(B)** Repertoire richness, measured as number of unique CDR3 sequences, normalized by number of total sequences. **(C)** Repertoire inequality, measured as Gini index of CDR3 sequence abundance distributions. **(D)** Repertoire diversity, measured as Shannon entropy. PLHIV, people living with HIV; HC, Healthy controls, p values for significant differences shown for Mann-Whitney U tests. Violin plots show distribution of data with individual data points, median and interquartile range. Alpha chain sequences are shown on the left and beta chain sequences on the right.

In TCR sequencing data derived from whole blood RNA, we interpreted unique alpha or beta chain sequences as surrogates for individual T cell clones. The number of total TCR sequences recovered for both alpha and beta chains were similar in PLHIV and controls (**Figure 1A**). However, three metrics which have been widely used to capture the TCR repertoire profile in a single number (reflecting both the number of distinct TCRs and their relative abundance) were significantly different among PLHIV and controls. First, repertoire richness (simply the total number of distinct alpha and beta chains in a sample independent of their relative abundance) was significantly reduced in PLHIV (**Figure 1B**). Second, the frequency distribution of alpha and beta TCR sequences was more skewed (showed greater inequality) in PLHIV, represented by a higher Gini index (**Figure 1C**). Third, the repertoire diversity captured by the Shannon entropy, which reflects the number of distinct TCRs but gives different weight to sequences on the basis of their abundance, was significantly lower in PLHIV (**Figure 1D**). These differences did not arise from any systematic differences in read depth, because they were still observed after randomly sub-sampling individual repertoires to the same number of total sequences (**Supplementary Figure 1**). None of these measures correlated with duration of therapy or pre-ART CD4 counts, but all were significantly associated with concurrent CD8 counts and the CD4:CD8 ratio (**Table 2**).

**Table 2:** Spearman correlation analyses of transcriptomic and TCR repertoire measurements with demographic and clinical parameters.

		Age^%^	Years ART^#^	CD4 count pre-ART^$^	CD4 count at sampling^#^	CD8 count at sampling^#^	CD4/CD8 at sampling^#^	EQ5D index^%^	SGRQ score^†^
**Modified MDP**									
	rho p-value	0.0512 0.76	0.0872 0.67	−0.1054 0.62	−**0.4390** **0.025**	0.1692 0.41	−0.3074 0.13	−0.0967 0.56	0.0101 0.96
**HIV module**									
	rho p-value	−0.0210 0.90	−0.3436 0.086	0.2663 0.20	−0.0359 0.86	−0.0133 0.95	−0.0137 0.95	−0.1142 0.49	−0.1758 0.34
**Richness**									
Alpha	rho p-value	−0.3031 0.064	−0.2636 0.19	0.1451 0.49	0.2681 0.19	−**0.4749** **0.014**	**0.4968** **0.0098**	0.2612 0.11	−0.0407 0.83
Beta	rho p-value	−0.2848 0.083	−0.2236 0.27	0.0851 0.69	0.2920 0.15	−**0.4304** **0.028**	**0.4773** **0.014**	0.2541 0.12	−0.0702 0.70
**Inequality**									
Alpha	rho p-value	0.2967 0.070	0.2423 0.23	−0.1516 0.47	−0.2906 0.15	**0.4715** **0.015**	−**0.4989** **0.0095**	−0.2661 0.11	0.0286 0.88
Beta	rho p-value	0.2754 0.094	0.2219 0.28	−0.0893 0.67	−0.3012 0.13	**0.4311** **0.028**	−**0.4811** **0.013**	−0.2536 0.12	0.0875 0.63
**Diversity**									
Alpha	rho p-value	−0.2418 0.14	−0.2309 0.26	0.2232 0.28	0.3833 0.053	−**0.4913** **0.011**	**0.6164** **0.0008**	0.2607 0.11	−0.0585 0.75
Beta	rho p-value	−0.1688 0.31	−0.1892 0.35	0.3090 0.13	**0.5102** **0.0078**	−0.2964 0.14	**0.5828** **0.0018**	0.2247 0.18	−0.0090 0.96
**Mean abundance top 100**									
Alpha	rho p-value	0.2075 0.21	0.0507 0.81	0.0089 0.97	−0.1104 0.59	**0.5337** **0.0050**	−**0.4238** **0.031**	−0.2509 0.13	−0.0088 0.96
Beta	rho p-value	0.1858 0.26	0.0193 0.93	0.0520 0.81	−0.1935 0.34	**0.4270** **0.030**	−0.3796 0.056	−0.1807 0.28	0.0396 0.83

Significant correlations (p-value < 0.05) are highlighted in bold. ^%^n = 38 (n = 26 PLHIV, n = 12 HC) **^#^**n = 26 (PLHIV) ^$^n = 25 (PLHIV) ^†^n = 32 (n = 22 PLHIV, n = 10 HC).

We tested the hypothesis that CD8 T cells were expanded in our long-term ART-treated PLHIV compared to controls, as previously reported (26, 27). Blood lymphocyte subset counts were not available in our HIV-negative control subjects for comparison to those of PLHIV. Instead, we used expression of validated transcriptional signatures for CD4- and CD8-positive T cells to compare their frequency in the different groups. We found comparable expression of the transcriptional CD4 T cell signature, but higher expression of the transcriptional CD8 T cell signature among PLHIV (**Supplementary Figure 2A**). Accordingly, the ratio of CD4:CD8 transcripts was lower in PLHIV compared to controls (**Supplementary Figure 2B**). To support the validity of this transcriptional analysis, we showed that CD4 and CD8 T cell counts correlated with their respective transcriptional signatures among PLHIV where flow cytometric quantitation of lymphocyte subsets was available (**Supplementary Figure 2C**). Likewise, the flow cytometric quantitation of the CD4:CD8 ratio correlated with the ratio of CD4:CD8 transcripts (**Supplementary Figure 2C**). Taken together, these data suggest that the persistent decrease in TCR repertoire diversity in PLHIV on long-term ART may be driven by oligoclonal expansion of CD8 T cells.

### Oligoclonal T Cell Expansion in People Living With HIV Is Unrelated to HIV, CMV or EBV

**Figure 2:**
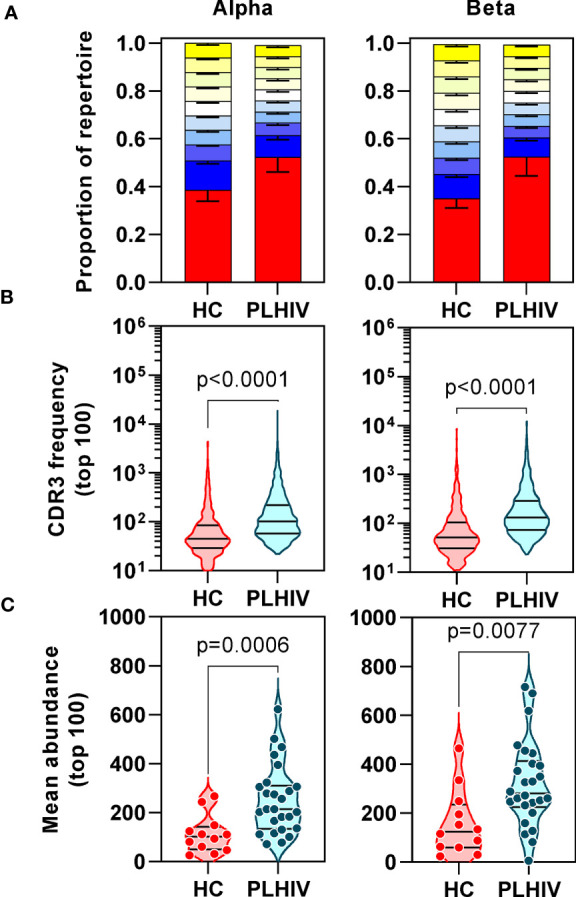
People living with HIV (PLHIV) show increased oligoclonal expansion of CDR3 sequences. **(A)** Frequency distribution of all CDR3 sequences, showing the proportion of the total repertoire that is occupied by each 10 percentile range of unique sequences. The percentile ranges are shown in decreasing order from the most abundant 10% (red) to the least abundant 10% (yellow at the top of each bar stack). Bars=median, error bars=interquartile range. **(B)** Frequency distribution of the 100 most abundant CDR3 sequences. **(C)** The mean abundance of the 100 most abundant CDR3 sequences in each individual.

**Figure 3:**
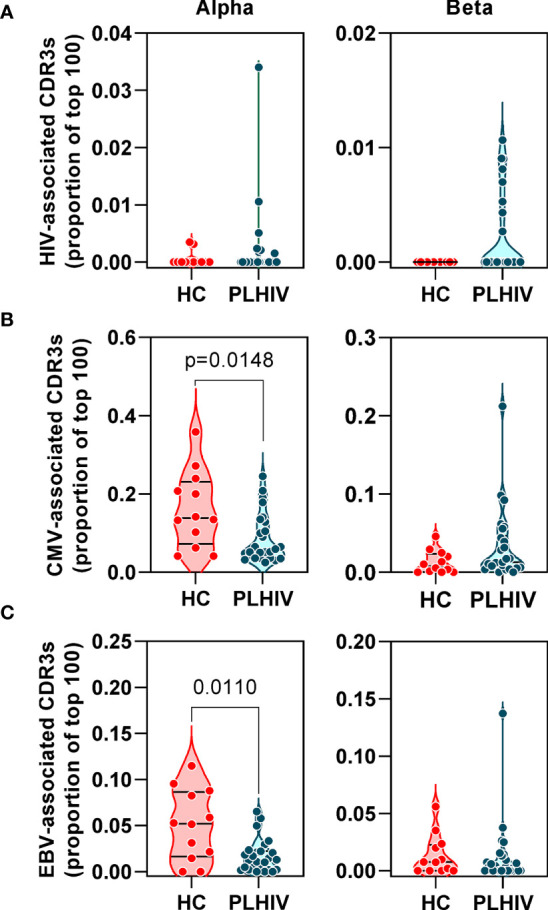
People living with HIV (PLHIV) do not show significant enrichment of HIV, CMV and EBV-reactive CDR3 sequences. Frequency of **(A)** HIV, **(B)** CMV and **(C)** EBV–reactive sequences, identified through public annotation on the VDJdb database, as a proportion of the total number of the 100 most abundant alpha and beta chain CDR3 sequences. PLHIV, people living with HIV; HC, Healthy controls; p values for significant differences shown for Mann Whitney U tests. Violin plots show distribution of data with individual data points, median and interquartile range.

We confirmed oligoclonal expansion of the TCR repertoire in long-term ART-treated PLHIV more directly by focusing on the most abundant CDR3 sequences. The 10% most common sequences occupied a significantly larger proportion of the total repertoire in PLHIV compared to controls (alpha: 52% vs 39%, Mann Whitney test p<0.0001; beta: 52% vs 35%, p=0.0003) (**Figure 2A**). Similarly, the frequency distribution of the 100 most abundant sequences was significantly shifted to larger clone sizes in PLHIV, with the mean abundance being two-fold greater compared to the controls (**Figures 2B, C**). The mean abundance of the 100 most common sequences was positively correlated with concurrent CD8 counts in PLHIV (**Table 2**), consistent with the hypothesis that CD8 T cells contribute to oligoclonal expansion within the TCR repertoire. We tested the hypothesis that the expanded clones in PLHIV may represent the response to persistent HIV despite effective suppression by ART, by counting the frequency of published HIV-reactive CDR3 sequences from the VDJ database of 20,433 alpha and 30,465 beta sequences (**Supplementary Table 1**) (44) in the pooled list of 100 most expanded CDR3 sequences from each group of study participants. Among PLHIV, 3/2311 (0.1%) alpha CDR3 sequences in 7/26 individuals and 7/2401 (0.3%) beta CDR3 sequences in 8/26 individuals were HIV reactive. This compared to 2/1067 (0.2%) alpha CDR3 sequences in 2/12 individuals and 0/1165 beta CDR3 sequences among controls (**Figure 3A**).

Oligoclonal CD8 T cell expansion is also a hallmark of CMV infection (58), with an even larger proportion of the memory CD8 T cell pool recognizing CMV in HIV-infected individuals compared to HIV-uninfected controls (59, 60). CMV serology was not available on all PLHIV and control subjects for which we had TCR sequence data. Instead, we used the TCR sequencing data to predict CMV serostatus as previously described (43). In this analysis, 87% of all the participants in our cohort were predicted to be CMV-positive with similar proportions in PLHIV and controls (**Table 1**). Based on the known CMV serology status in a subset of our PLHIV group (n=13), the overall accuracy of this classification approach was 85% (10/11 CMV-positive and 1/2 CMV-negative individuals being correctly identified). In view of the high prevalence for CMV positivity among our cohort, we evaluated the frequency of CMV-reactive CDR3 sequences from VDJdb as described above for HIV-reactive sequences. Among PLHIV, 161/2311 (7%) alpha CDR3 sequences and 54/2401 (2.2%) beta CDR3 sequences were CMV-reactive. This compared to 94/1067 (0.2%) alpha CDR3 sequences and 20/1165 (1.7%) beta CDR3 sequences among controls. Neither alpha nor beta chain CMV–associated sequences were enriched in PLHIV compared to controls (**Figure 3B**). Similarly, extension of this analysis to another prevalent herpes virus, EBV, failed to show any virus-specific CDR3 sequence enrichment among PLHIV (**Figure 3C**). Taken together, these data reject the hypothesis that oligoclonal T cell expansion in PLHIV is due to HIV, CMV or EBV-associated responses.

### Reduced Intra-Individual Similarity of CDR3 Sequences in People Living With HIV

**Figure 4:**
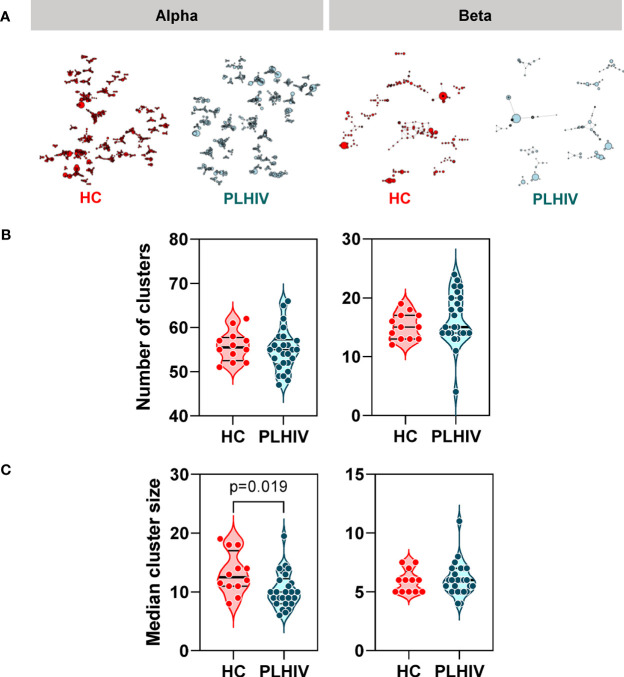
Reduced intra-individual similarity of CDR3 sequences in people living with HIV (PLHIV). **(A)** Network graphs showing clusters of related alpha or beta chain CDR3 sequences from representative repertoires of healthy controls (HC) or PLHIV. Networks were created from the 2500 most abundant CDR3 sequences in each repertoire. Each node represents a unique CDR3 sequence, with the node diameter proportional to its abundance in the repertoire. Two CDR3 nodes are connected by an edge if they differ from each other by a Levenshtein distance of one. Only clusters with four or more nodes are shown. **(B)** Number of alpha or beta chain CDR3 clusters in each individual. **(C)** Median cluster size (number of nodes) for alpha or beta chain CDR3 clusters in each individual. p values for significant differences shown for Mann Whitney U tests. Violin plots show distribution of data with individual data points, median and interquartile range.

In order to test whether specific or related antigens other than HIV, CMV or EBV may drive oligoclonal expansion of T cell clones in PLHIV compared to controls, we measured intra-individual CDR3 amino acid sequence similarity on the premise that TCRs with more similar CDR3s are more likely to recognize related antigens (61). We measured similarity between CDR3 pairs within an individual, using the Levenshtein distance (the minimum number of single-character edits required to turn one string into another) (37). To reduce computational time, we restricted this analysis to the 2500 most abundant CDR3 sequences. For each individual we constructed a similarity network connecting CDR3 sequences (nodes) that differed by a Levenshtein distance of one (**Figure 4A**). In this analysis, the convergence of TCR clones into fewer but larger clusters would suggest that the repertoire may be targeting the same or related antigens. In fact, the number of clusters was equivalent in PLHIV and controls, and the cluster sizes were smaller in PLHIV among alpha chain sequences (**Figures 4B, C**). In a sensitivity analysis, we found similar results when the analysis was restricted to CDR3 sequences that were present at least three times in an individual, and more likely to be derived from memory T cells (40) (**Supplementary Figures 3B, C**). These data suggest that expanded clones in long-term ART treated PLHIV do not arise as a result of reactivity to a common target, but may represent expansion of a divergent population of T cells directed at a heterogenous set of antigens in each individual.

### People Living With HIV Have More T Cell Receptors With Unusually Long CDR3 Sequences

**Figure 5:**
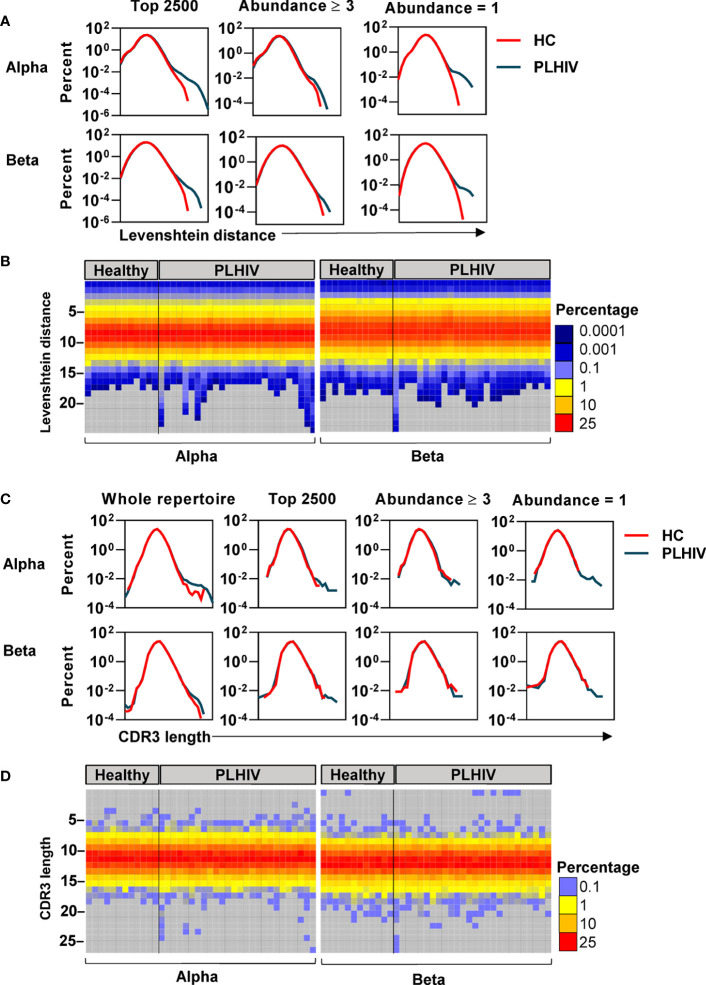
A subset of people living with HIV (PLHIV) have more T cell receptors (TCRs) with unusually long CDR3 sequences. **(A)** Frequency distributions of Levenshtein distances among alpha and beta CDR3 amino acid sequences, integrated across all PLHIV or all healthy controls (HC). Pairwise Levenshtein distances were calculated for the 2500 most abundant CDR3s in each repertoire, or restricted to CDR3s that were present at least three times (the 980 most abundant sequences in each repertoire) or only once (the 980 least abundant sequences in each repertoire). One beta sample from the PLHIV group was excluded from the analysis of the top 980 sequences as it contained <100 CDR3s with abundance ≥3. **(B)** Heatmaps of Levenshtein distance distributions among the least abundant sequences as defined in **(A)**. Each column represents the repertoire from an individual subject. **(C)** Frequency distributions of CDR3 amino acid sequence lengths among alpha and beta chains, integrated across all PLHIV or HC. Length distributions were determined for the whole repertoire, or restricted to a subset of CDR3 sequences as defined in **(A)**. **(D)** Heatmaps of CDR3 length distributions among the least abundant sequences.

To further investigate the mechanisms that contribute to the greater dissimilarity of CDR3 sequences within individual PLHIV, we compared the frequency distributions of Levenshtein distances across PLHIV and controls. The integrated data from PLHIV showed a population of highly dissimilar CDR3 sequences with Levenshtein distances >20 that were not present in controls (**Figure 5A**).

CDR3s with a clone size of ≥3 are most likely to represent memory T cells, whereas those that are present only once are most likely to represent naïve T cells (40). The highly dissimilar CDR3s in PLHIV were only evident in the least abundant sequences, suggesting that they were a feature of naïve T cells (**Figure 5A**). At the individual level, this observation was not evident in every case, but more pronounced in a subset of PLHIV (**Figure 5B**). The average CDR3 length of TCRs is 13–15 amino acids (62). Therefore, we hypothesized that such large Levenshtein distances of >20 reflect the presence of TCRs with unusually long CDR3 sequences in PLHIV. We evaluated this hypothesis both on the level of nucleotide and amino acid sequences. PLHIV had marginally more alpha and beta TCRs with long (>40) nucleotide inserts at the VJ gene junction, while the number of nucleotide deletions from the V and J genes (relative to their germline sequences) was comparable to controls (**Supplementary Figure 4**). However, some PLHIV showed more alpha and beta TCRs with very long CDR3 sequences (>20 amino acids) (**Figures 5C, D**). Consistent with the analysis of Levenshtein distances, these findings were more evident in the least abundant clones likely to represent naïve T cells. Such sequences are thought to be restricted during normal thymic selection (63). Therefore, their presence in peripheral blood of PLHIV may reflect thymic dysfunction.

### Reduced Inter-Individual Sharing of Identical CDR3 Sequences in People Living With HIV

**Figure 6:**
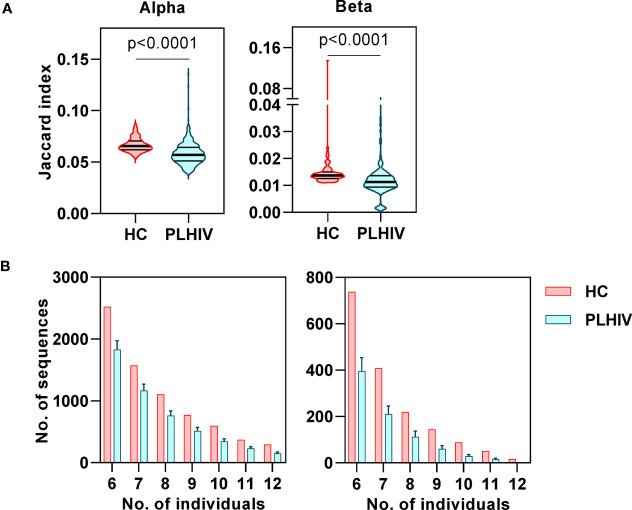
Reduced inter-individual sharing of identical CDR3 sequences in people living with HIV (PLHIV). **(A)** The proportion of identical CDR3 sequences that are shared between repertoires of each pair of healthy controls (HC) or PLHIV, calculated as Jaccard index. p values for significant differences shown for Mann Whitney U tests. Violin plots show distribution of data with median and interquartile range. **(B)** Frequency distributions showing the number of identical alpha or beta CDR3 sequences that are found in the repertoires of 6 to 12 out of 12 HC (red) or PLHIV (blue). To correct for the larger size of the PLHIV cohort (n=26 versus n=10 HC), the plots show the average results (mean+SD) of analyzing 100 random samples of 12 out of the 26 PLHIV samples available.

In our previous study of untreated and short-term treated HIV disease, we had found a reduction in the number of ‘public’ CDR3 sequences (public being defined as the number of sequences shared by more than one individual) (37). Interestingly, public sequences have also recently been reported to be dependent on normal thymic selection (64). In view of the evidence of potential thymic dysfunction among PLHIV described above, we tested the hypothesis that long-term ART treated PLHIV show a persistent reduction in public TCRs. Using the Jaccard index to calculate the overlap of identical CDR3s between pairs of PLHIV, we found that inter-individual sharing of both alpha and beta chain sequences remained significantly decreased in PLHIV compared to controls (**Figure 6A**). Similarly, the number of CDR3 sequences that were highly public (found in at least 50% of individuals) was substantially reduced among the group of PLHIV (**Figure 6B**).

### Persistent T Cell Activation in People Living With HIV on Long-Term Anti-Retroviral Therapy

**Figure 7:**
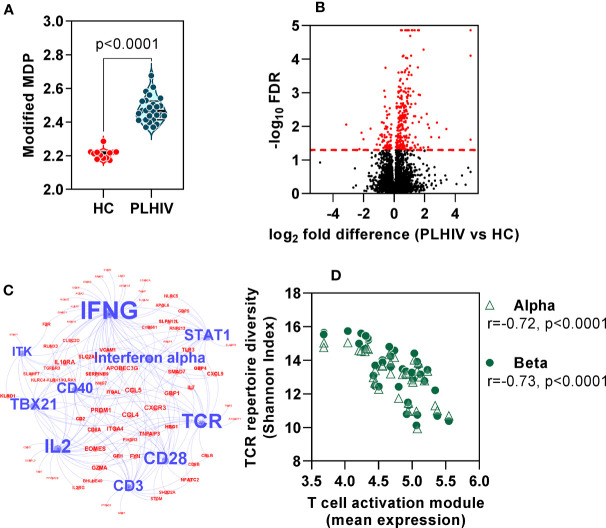
Global immune activation at steady state in people living with HIV (PLHIV) despite effective anti-retroviral therapy. **(A)** Modified molecular degree of perturbation (MDP) of healthy controls (HC) and PLHIV. p values for significant differences shown for Mann Whitney U tests. Violin plots show distribution of data with individual data points, median and interquartile range. **(B)** Volcano plot showing statistical significance against quantitative gene expression differences between PLHIV and HC. The red dashed line indicates a false discovery rate (FDR) of 0.05, equivalent to -log_10_ FDR of 1.3. Genes highlighted in red are considered differentially expressed (n=353; n=281 higher in PLHIV and n=72 higher in HC). **(C)** Network diagram showing predicted upstream regulators of interacting differentially expression genes (see **Supplementary Figure 5**), found to be enriched in PLHIV. Blue nodes represent the ten most significant upstream regulators, with label size proportional to the -log_10_ enrichment p value. Red nodes represent the subset of the 149 interacting genes that are downstream of these regulators. **(D)** Spearman rank correlation of TCR diversity (Shannon Index) and mean expression of target genes (red nodes) in **(C)**, among all participants.

In order to evaluate the functional significance of the persistent changes in the T cell repertoire among PLHIV, we looked for evidence of associated immune dysfunction at systems level, by whole blood genome-wide transcriptional profiling of the same samples. PLHIV had significantly greater perturbation of blood transcriptional profiles compared to control subjects (**Figure 7A**), as measured by the modified molecular degree of perturbation (MDP) (50), representing the extent to which each individual transcriptome deviated from the mean of control subjects as a standard reference. Next, we assessed the number of differentially expressed genes (DEG) between the two groups (FDR <0.05, **Figure 7B**). Of 353 DEG, 281 genes were higher in PLHIV. We hypothesized that these would reflect enrichment of specific immunological pathways which represented differences in functional immunological profiles at steady state. To evaluate these pathways, we first identified a subset of 149 genes that are predicted to interact directly or indirectly, thereby reflecting biological systems. This interactome showed enrichment of immune response pathways (**Supplementary Figure 5**, **Supplementary File 3**). Upstream regulator analysis revealed the interactome to represent molecules primarily involved in T cell activation and interferon (IFN)γ signaling (**Figure 7C**, **Supplementary File 3**). Together, these data suggest persistently elevated T cell activation in PLHIV on long-term ART. Importantly, the mean expression of the genes representing these systems correlated with the reduction in diversity of the TCR repertoires (**Figure 7D**).

### Anti-Retroviral Therapy Attenuates or Resolves Gene Signatures Associated With Untreated HIV Infection

**Figure 8:**
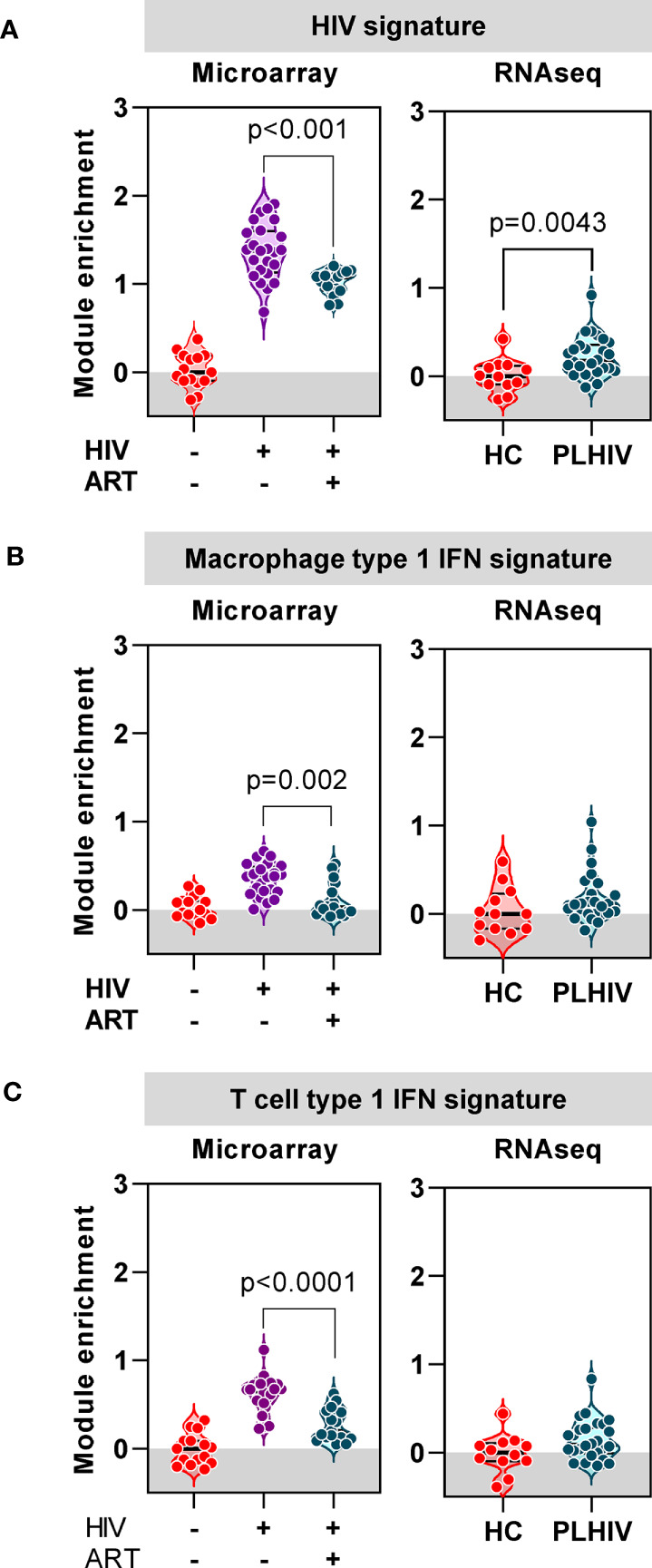
Anti-retroviral therapy (ART) attenuates or resolves the profound impact of chronic HIV infection on transcriptional signatures. **(A)** Mean expression of a gene signature (module) associated with untreated HIV derived from microarray data, in blood from a previous cohort of HIV-negative patients, and HIV-positive patients before and 3 months after ART (left panel), and from RNAseq data in blood from healthy controls (HC) and PLHIV described in the present study (right panel). **(B)** Mean expression of a type-1 interferon (IFN) inducible gene signature (module) derived from monocyte derived macrophages, in blood from a previous cohort of HIV-negative patients, and HIV-positive patients before and 3 months after ART (left panel), and from RNAseq data in blood from healthy controls (HC) and PLHIV described in the present study (right panel). **(C)** Mean expression of a type-1 interferon (IFN) inducible gene signature (module) derived from T cells, in blood from a previous cohort of HIV-negative patients, and HIV-positive patients before and 3 months after ART (left panel), and from RNAseq data in blood from healthy controls (HC) and PLHIV described in the present study (right panel). p values for significant differences shown for Mann Whitney U tests. Violin plots show distribution of data with individual data points, median and interquartile range.

Finally, we evaluated the extent to which the blood transcriptional findings in long–term ART-treated PLHIV were distinct from those that are associated with untreated HIV. We took advantage of independent microarray transcriptomic data from our previous longitudinal case-control study of TCR repertoires in HIV-infected individuals before and after three months ART compared to HIV-negative controls (37). In this dataset, we observed a large number of DEG that were enriched in untreated HIV patients compared to HIV-negative controls (4542 of 9292 DEG). In addition to a statistical cut-off (FDR <0.05), we applied a fold change filter of ≥2 to derive a signature of 434 transcripts that were most increased in untreated HIV (**Supplementary Figure 6A**, **Supplementary File 1**). Consistent with previous reports on transcriptional changes in untreated HIV (65–67), this signature was enriched for immune- and cell cycle-associated pathways, and dominated by type 1 IFN signaling (**Supplementary Figures 6B, C**, **Supplementary File 1**). There was significant but partial reversal of the expression of this untreated HIV-associated signature after three months ART (**Figure 8A**). In RNAseq data from our cohort of PLHIV on long-term ART, the expression of this transcriptional signature overlapped with that of HIV-negative controls, albeit with a distribution that remained statistically higher (**Figure 8A**).

In view of the prediction from our bioinformatic analysis that the untreated HIV signature was primarily driven by type 1 IFN signaling, we extended our analysis to evaluate the expression of two separate type 1 IFN-inducible transcriptional signatures independently derived from macrophages (53) and T cells (**Supplementary File 2**), respectively. The expression of both these signatures was significantly enriched in untreated PLHIV, partially normalized in response to three months of ART, and comparable to HIV-negative controls in PLHIV on long-term ART (**Figures 8B, C**). Taken together, our analysis of blood transcriptomic data suggests that long-term ART in PLHIV leads to resolution of elevated type 1 IFN activity associated with untreated infection, but increased levels of T cell activation-associated IFNγ activity.

## Discussion

We present the first paired TCR repertoire sequencing and global transcriptomic profiling of blood samples from PLHIV. Similar to our previous analysis of untreated or short-term ART-treated HIV (37), PLHIV on long-term ART still showed significantly less TCR repertoire diversity compared to HIV-negative controls, underpinned by oligoclonal expansion of T cell clones. Based on evidence for a persistently elevated CD8 T cell fraction in the peripheral blood of long-term ART-treated PLHIV, we hypothesise that CD8 T cells are responsible for the persistent changes in the TCR repertoire. We hypothesise that increased clonal expansions are unlikely to be driven by HIV antigens themselves, since HIV levels remain undetectable in PLHIV. They also cannot be explained by differences in CMV reactivity that has otherwise been reported to drive large proportions of CD8 T cell responses (58–60). Identifying the targets of the expanded T cells will be important in trying to understand the underlying pathology associated with PLHIV. In addition, two lines of evidence suggest that persistent abnormalities of the TCR repertoire in long-term ART-treated PLHIV may reflect dysfunctional thymic output. First, we found increased frequency of long CDR3 sequences among the least abundant clones, most likely to represent the naïve T cell fraction, that are typically deleted in normal thymic selection (63). Secondly, we found reduced frequency of public TCR sequences that are thought to depend on normal thymic selection (64).

We complemented our repertoire analysis with evidence of T cell dysfunction in PLHIV by bioinformatic analysis of genome-wide transcriptomes. We found that the striking perturbation of the blood transcriptome in untreated HIV, reflecting an exaggerated type 1 IFN response, was largely resolved in long-term ART-treated PLHIV. Nonetheless, the blood transcriptome remained consistently abnormal in these patients with changes in sets of genes linked to increased T cell activation which were significantly correlated to changes to the TCR repertoire. The present study is limited to white European adult males. Future extension to more diverse demographic groups is required to confirm its generalizability. In addition, we have not established the activation phenotype or the antigen specificity of the expanded T cells we observe in PLHIV. Single cell sequencing analysis will be needed to address this question, and to validate our application of unique alpha and beta sequences as surrogates for T cell clones. The possibility that they may react to microbial antigens arising from translocation of gastrointestinal products, or perhaps abnormally presented self-antigens, offers plausible alternative hypotheses to test. We were also not able to determine the relative contribution of oligoclonal expansion of CD8 T cells and abnormal recovery of the CD4 T cell repertoire.

Our data are consistent with a model in which thymic dysfunction may lead to repopulation of the T cell repertoire with clones that have greater propensity for functional dysregulation, for example as a result of autoreactivity or abnormal MHC restriction and manifest in the peripheral blood transcriptome with evidence of increased T cell activation. Such a model represents an important paradigm shift in our understanding of the mechanisms of chronic immune activation among PLHIV. In view of the relationship between chronic immune activation and adverse clinical outcomes in long-term ART-treated PLHIV, answers to these questions may inform novel therapeutic approaches to restore normal immune function in HIV infection and further reduce chronic morbidity in this population.

## Data Availability** Statement

RNAseq data are available at Array Express E-MTAB-8891 (https://www.ebi.ac.uk/arrayexpress). Microarray data are available at Array Express E-MTAB-8897 (https://www.ebi.ac.uk/arrayexpress). TCR sequencing data are available at NCBI Short Read Archive accession number PRJNA613091 (https://www.ncbi.nlm.nih.gov/sra).

## Ethics Statement

The studies involving human participants were reviewed and approved by the London Hampstead Research Ethics Committee. The patients/participants provided their written informed consent to participate in this study.

## Author Contributions

JB, ML, BC, and MN conceived and designed the study. JB, ES-W, IU, ET, and JR acquired the data. CT led the data analysis supported by JB, GP, YS, BC, and MN. CT and MN wrote the manuscript with input from all the authors. All authors contributed to the article and approved the submitted version.

## Funding

This project was supported by Wellcome Trust funding to MN (207511/Z/17/Z), Rosetrees Trust (M824) and National Institute for Health Research (DRF-2015-08-210) funding to JB, and NIHR Biomedical Research Funding to UCL and UCLH.

## Conflict of Interest

The authors declare that the research was conducted in the absence of any commercial or financial relationships that could be construed as a potential conflict of interest.

## Supplementary Material

Supplementary File S1 Untreated HIV module gene composition, and all upstream regulators and Reactome pathways of the constituent 434 genes.

Supplementary File S2 Constituent genes of the T cell type 1 IFN module.

Supplementary File S3 All upstream regulators and Reactome pathways of the 149 genes that are significantly up-regulated in people living with HIV versus healthy controls and known to interact with each other.

Supplementary Data Sheet Supplementary Figures 1–6 and Supplementary Table 1.
