# Supplementary material for: Persistent T Cell Repertoire Perturbation and T Cell Activation in HIV After Long Term Treatment
Source: Front Immunol. 2021 Feb 25;12:634489. doi: 10.3389/fimmu.2021.634489 (PMC7959740; doi:10.3389/fimmu.2021.634489)
Supplement: Supplementary Data Sheet — Supplementary Figures 1–6 and Supplementary Table 1. [file DataSheet_1.pdf]

Persistent T cell repertoire perturbation and T cell activation in HIV after long term treatment- Supplementary data

Persistent T cell repertoire perturbation and T cell activation in HIV after long term treatment- Supplementary data 1
Supplementary Figure 1 2
Subsampled T cell receptor alpha and beta chain sequence repertoires 2
Supplementary Figure 2 3
People living with HIV (PLHIV) show higher expression of a transcriptional CD8 T cell signature, and lower CD4:CD8 transcript ratio, compared to healthy controls (HC). 3
Supplementary Figure 3 4
Reduced intra-individual similarity of expanded CDR3 sequences in people living with HIV (PLHIV). 4
Supplementary Figure 4 5
Junctional nucleotide diversity is comparable between people living with HIV (PLHIV) and healthy controls (HC). 5
Supplementary Figure 5 6
Interactome and reactome pathway analysis of genes significantly upregulated in people living with HIV (PLHIV). 6
Supplementary Figure 6 7
Untreated HIV blood transcriptional signature. 7
Supplementary Table 1 8

Supplementary Figure 1

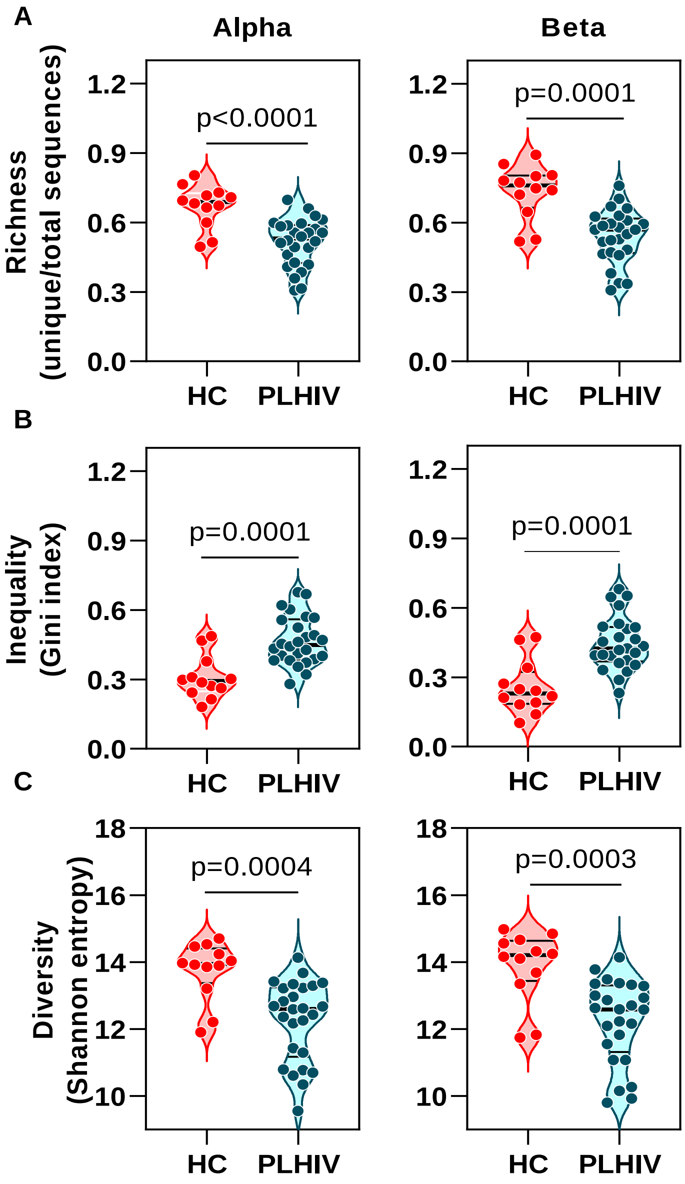

Subsampled T cell receptor alpha and beta chain sequence repertoires

Repertoires were 100 times randomly subsampled to the same number of total CDR3s (40,000 alpha or beta sequences). Data are shown for **(A)** richness, **(B)** inequality and **(C)** diversity for alpha and beta chain sequences among healthy controls (HC) and PLHIV. One beta sample was omitted as its total sequence count was <3000. p values for significant differences shown for Mann-Whitney U tests. Violin plots show distribution of data with individual data points, median and interquartile range.

Supplementary Figure 2

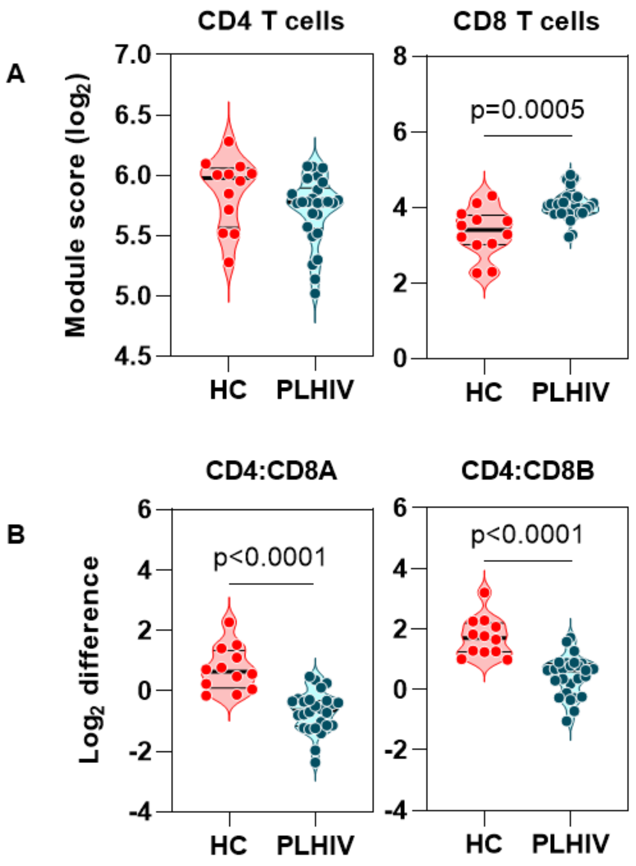

C

	CD4 module	CD8 module	CD4:CD8A gene ratio	CD4:CD8B gene ratio
**Correlation with blood CD4 count**				
Rho	0.7133	0.1228	0.1723	-0.0003419
P	**<0.0001**	0.55	0.40	1.00
**Correlation with blood CD8 count**				
Rho	0.1453	0.5918	-0.6499	-0.7299
P	0.48	**0.0014**	**0.0003**	**<0.0001**
**Correlation with blood count CD4:CD8 ratio**				
Rho	0.3193	-0.4574	0.7733	0.6969
P	0.11	**0.019**	**<0.0001**	**<0.0001**

People living with HIV (PLHIV) show higher expression of a transcriptional CD8 T cell signature, and lower CD4:CD8 transcript ratio, compared to healthy controls (HC).

(A) Mean expression of blood transcriptional signatures (modules) representing CD4 and CD8 T cells, **(B)** ratio of CD4:CD8A and CD4:CD8B expression in blood, calculated as difference in log_2 transcript per million (TPM), from healthy controls and PLHIV. **(C)** Spearman correlation analyses of transcriptional CD4 and CD8 measurements with lymphocyte subset cell counts in blood from PLHIV (n=26). p values for significant differences shown for Mann-Whitney U tests. Violin plots show distribution of data with individual data points, median and interquartile range. Significant correlations in bold.

Supplementary Figure 3

Reduced intra-individual similarity of expanded CDR3 sequences in people living with HIV (PLHIV).

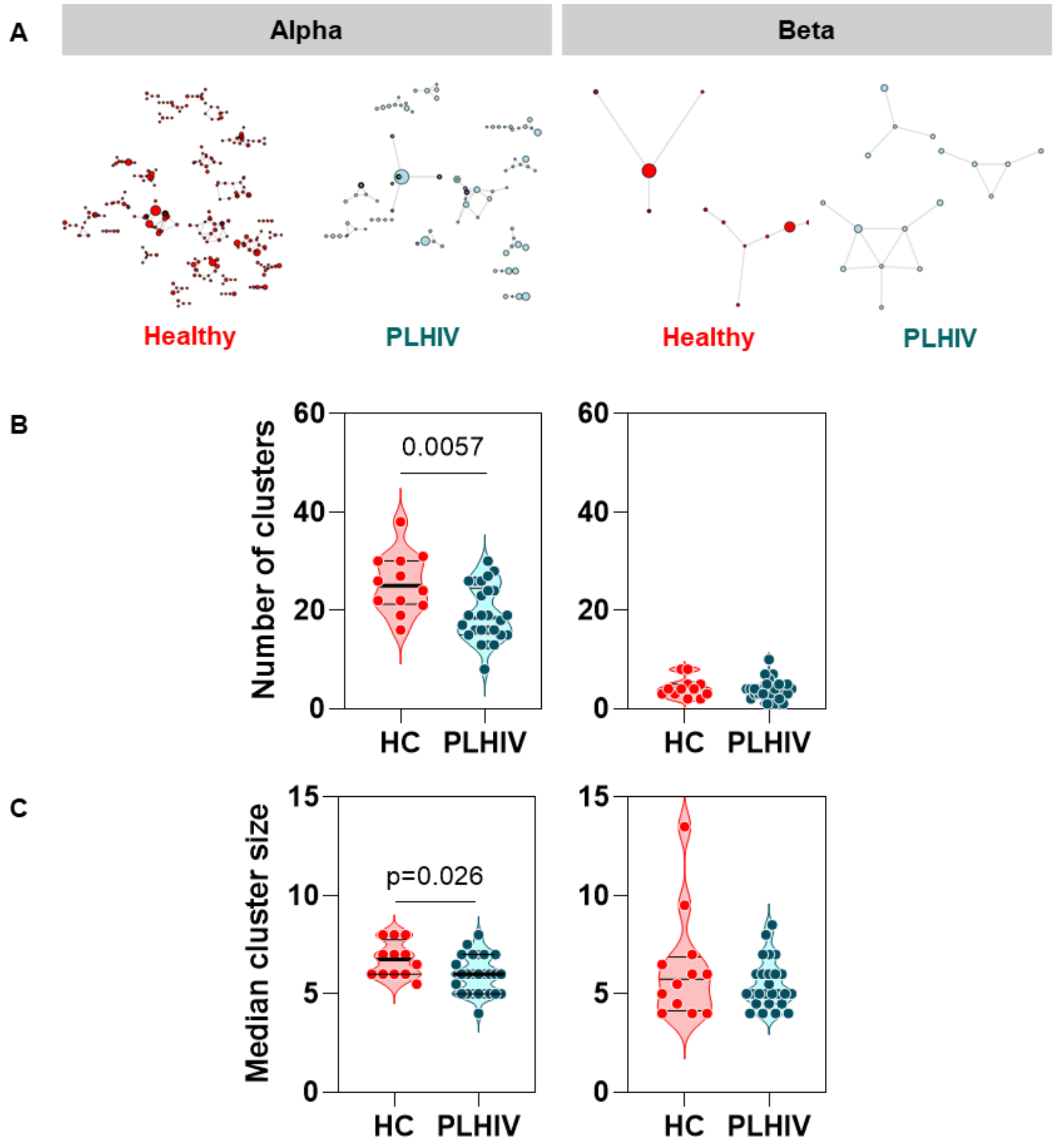

(A) Network graphs showing clusters of related alpha or beta chain CDR3 sequences from representative repertoires of healthy controls (HC) or PLHIV. Networks were created from the 980 most abundant CDR3 sequences in each repertoire, and thus restricted to CDR3s that occurred at least three times. Each node represents a unique CDR3 sequence, with the node diameter proportional to its abundance in the repertoire. Two CDR3 nodes are connected by an edge if they differ from each other by a Levenshtein distance of one. Only clusters with four or more nodes are shown. **(B)** Number of alpha or beta chain CDR3 clusters in each individual and **(C)** median cluster size (number of nodes) for alpha or beta chain CDR3 clusters in each individual. p values for significant differences shown for Mann Whitney U tests. Violin plots show distribution of data with individual data points, median and interquartile range.

Supplementary Figure 4

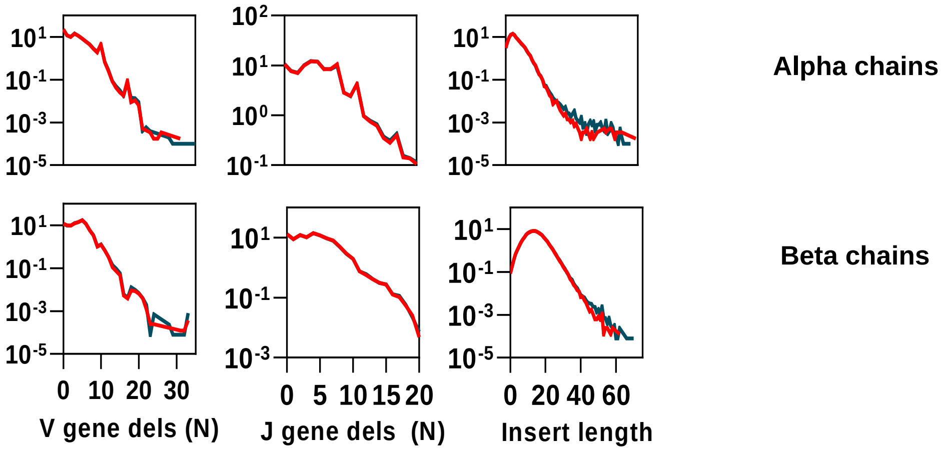

Junctional nucleotide diversity is comparable between people living with HIV (PLHIV) and healthy controls (HC).

Frequency distributions show the number of V gene deletions, J gene deletions, and nucleotide insert length of alpha or beta T cell receptor sequences, integrated across all PLHIV (black lines) or all HC (red lines), and assessed on the level of the whole repertoire.

Supplementary Figure 5

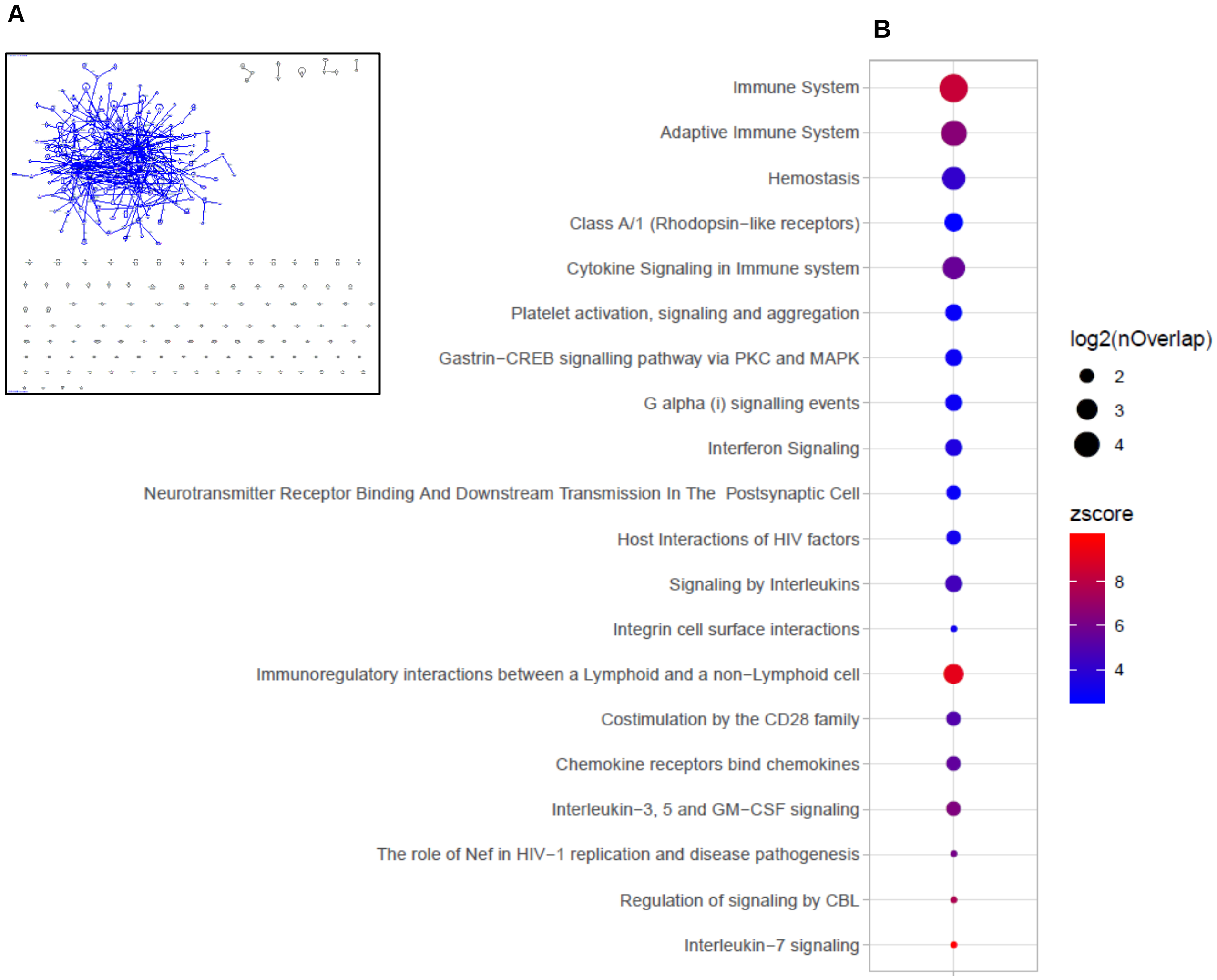

Interactome and reactome pathway analysis of genes significantly upregulated in people living with HIV (PLHIV).

(A) Network of 149 directly and indirectly interacting genes among 281 genes that are significantly higher expressed in PLHIV compared to HC, from those identified in Figure 7B in main manuscript. **(B)** Heatmap of enriched Reactome pathways among 149 interacting genes that are upregulated in PLHIV. Bubble size indicates the number of genes among the 149 interacting genes that are mapped to each pathway. Bubble colour represents the enrichment z-score.

Supplementary Figure 6

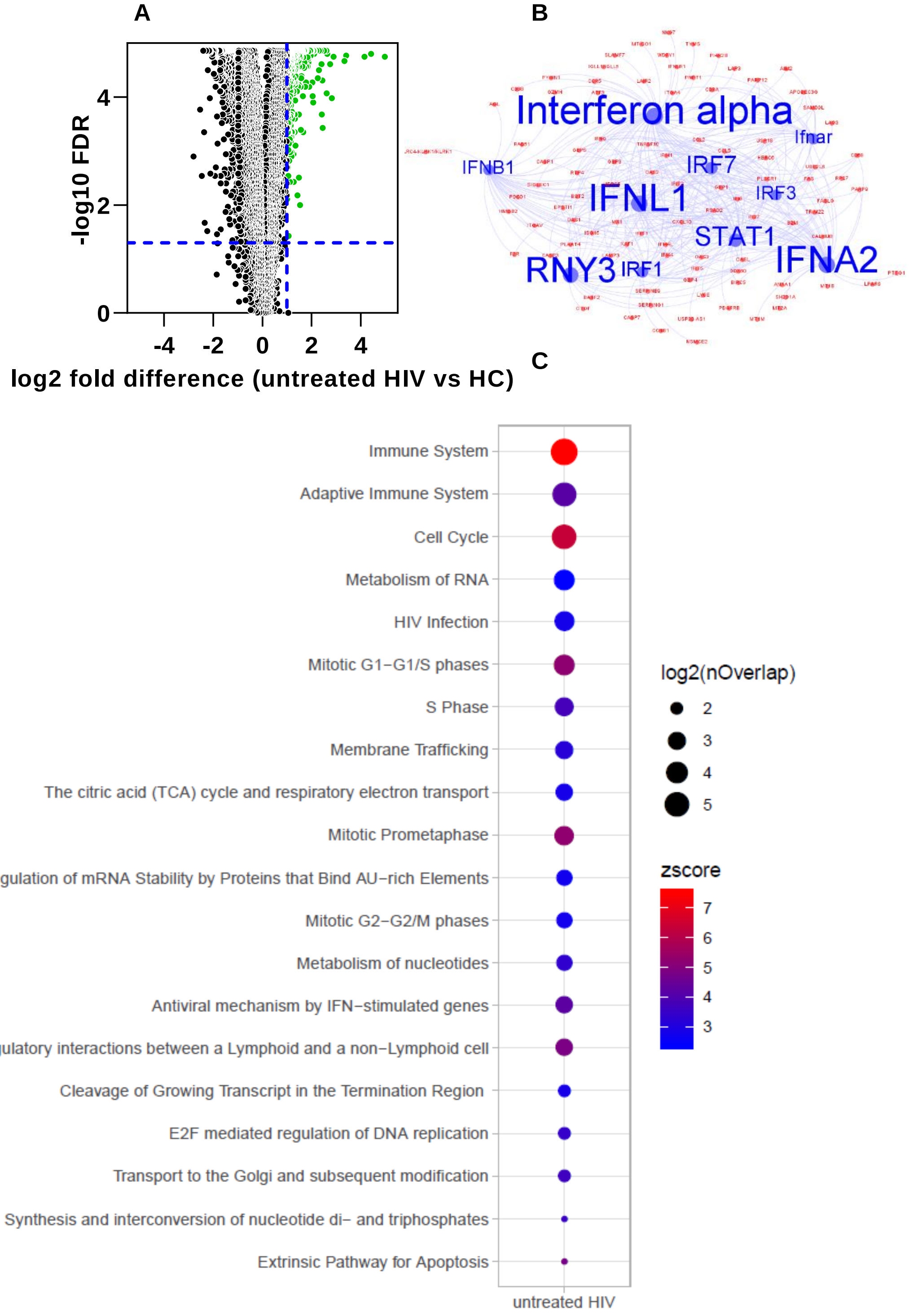

Untreated HIV blood transcriptional signature.

(A) Volcano plot shows statistical significance against gene expression differences between HC and untreated HIV patients. The blue dashed lines indicate a false discovery rate (FDR) of 0.05, equivalent to $-\log_{10}$ FDR of 1.3, and a fold change of 2, equivalent to $\log_2$ fold difference of 1. Genes highlighted in green were included in the untreated HIV signature (n=434). **(B)** Heatmap of enriched Reactome pathways in the untreated HIV signature. Bubble size indicates the number of genes among the 434 signature genes that are mapped to each pathway. Bubble colour represents the enrichment z-score. **(C)** Network diagram shows predicted upstream regulators of the untreated HIV signature genes. Blue nodes represent the ten most significant upstream regulators with activation z-scores >2, with node size proportional to the $-\log_{10}$ enrichment p value. Red nodes represent the subset of the 434 signature genes that act downstream of these regulators.

Supplementary Table 1

	Full dataset	HIV-1 reactive	CMV reactive	EBV reactive
**Alpha chain sequences**	20,433	304	14,036	3,024
**Beta chain sequences**	30,465	2,093	17,872	4,239

Number of human CDR3 sequences in the full VDJ database (https://vdjdb.cdr3.net/; accessed 26/11/2019) (44) and associated with reactivity to HIV, CMV and EBV.
